# SERPINE2 Inhibits IL-1α-Induced MMP-13 Expression in Human Chondrocytes: Involvement of ERK/NF-κB/AP-1 Pathways

**DOI:** 10.1371/journal.pone.0135979

**Published:** 2015-08-25

**Authors:** Anna Santoro, Javier Conde, Morena Scotece, Vanessa Abella, Ana Lois, Veronica Lopez, Jesus Pino, Rodolfo Gomez, Juan J. Gomez-Reino, Oreste Gualillo

**Affiliations:** 1 SERGAS (Servizo Galego de Saude) and IDIS (Instituto de Investigación Sanitaria de Santiago), the NEIRID Lab (Neuroendocrine Interactions in Rheumatology and Inflammatory Diseases), Research Laboratory 9, Santiago University Clinical Hospital, Santiago de Compostela, Spain; 2 Department of Molecular and Cellular Biology, University of Coruña (UDC), A Coruña, Spain; 3 University of Naples Federico II, Dept. of Pharmacy, 80138, Naples, Italy; 4 SERGAS (Servizo Galego de Saude), Division of Orthopaedics Surgery and Traumatology, Santiago University Clinical Hospital, Santiago de Compostela, Spain; 5 University of Santiago de Compostela, Department of Medicine and SERGAS (Servizo Galego de Saude) and IDIS (Instituto de Investigación Sanitaria de Santiago), Division of Rheumatology, Santiago University Clinical Hospital, Santiago de Compostela, Spain; Queen Mary University of London, UNITED KINGDOM

## Abstract

**Objectives:**

Osteoarthritis (OA) is a chronic joint disease, characterized by a progressive loss of articular cartilage. During OA, proinflammatory cytokines, such as interleukin IL-1, induce the expression of matrix metalloproteinases (MMPs) in chondrocytes, contributing thus to the extracellular matrix (ECM) degradation. Members of Serpine family, including plasminogen activator inhibitors have been reported to participate in ECM regulation. The aim of this study was to assess the expression of serpin peptidase inhibitor clade E member 2 (SERPINE2), under basal conditions and in response to increasing doses of IL-1α, in human cultured chondrocytes. We also examined the effects of SERPINE2 on IL-1α-induced MMP-13 expression. For completeness, the signaling pathway involved in this process was also explored.

**Methods:**

SERPINE2 mRNA and protein expression were evaluated by RT-qPCR and western blot analysis in human T/C-28a2 cell line and human primary chondrocytes. These cells were treated with human recombinant SERPINE2, alone or in combination with IL-1α. ERK 1/2, NFκB and AP-1 activation were assessed by western blot analysis.

**Results:**

Human cultured chondrocytes express SERPINE2 in basal condition. This expression increased in response to IL-1α stimulation. In addition, recombinant SERPINE2 induced a clear inhibition of MMP-13 expression in IL-1α-stimulated chondrocytes. This inhibitory effect is likely regulated through a pathway involving ERK 1/2, NF-κB and AP-1.

**Conclusions:**

Taken together, these data demonstrate that SERPINE2 might prevent cartilage catabolism by inhibiting the expression of MMP-13, one of the most relevant collagenases, involved in cartilage breakdown in OA.

## Introduction

Osteoarthritis (OA) is one of the most common rheumatic disorders and a major cause of pain and disability in older adults. Although, OA is considered a primary disorder of articular cartilage, it is generally accepted that OA is a disease of the whole joint, and other tissues, such as synovia, bone and ligaments, are also affected [1].

Chondrocytes, the unique cell type of adult articular cartilage, remain as quiescent cells in normal conditions, maintaining the turnover of the extracellular matrix components. However, during OA, chondrocytes become activated, characterized by phenotypic changes and increased production of extracellular matrix-degrading enzymes, which include different matrix metalloproteinases (MMPs) [2]. This chondrocyte-phenotypic shift is caused, in part, by the exposure to abnormal environmental insults, including high mechanical stress, metabolic alterations, pro-inflammatory cytokines and adipokines [2–4]. One of the most relevant pro-inflammatory cytokines involved in cartilage degeneration is interleukin-1 (IL-1). In fact, this cytokine is able to induce MMP-13, one of the most relevant collagenases involved in cartilage breakdown during OA.

Serine proteases play a predominant role in multiple systems and they are regulated by a large family of structurally related proteins called serpins. One of the better-known and most studied members of serpine superfamily is plasminogen activator inhibitor 1 (PAI-1 or SERPINE1). The expression of PAI-1, which regulates plasmin activation, was found to be significantly decreased in OA cartilage as compared to healthy controls [5]. In addition, it has been reported that the plasminogen activator/inhibitor system participated in the regulation of the cartilage extracellular matrix (ECM) homeostasis and in the OA pathophysiology process [5,6]. Actually, OA cartilage has been shown elevated levels of urokinase plasminogen activator (uPA) and tissue-type plasminogen activator (tPA), as well as increased plasmin activity [5], which are related with degradation processes and MMPs activation [7,8].

SERPINE2 (serpin peptidase inhibitor clade E member 2), also known as protease nexin-1 (PN-1), belongs to serpin superfamily. It is the closest relative of PAI-1, and it can inhibit the activities of different proteases including thrombin, urokinase, trypsin or plasmin [9]. This 43 kDa glycoprotein is encoded by the *SERPINE2* gene on human chromosome 2q99-q35.6 [10] and its expression was identified in many mouse tissues, such as brain, kidney, lung, spleen, muscle and cartilage [11].

SERPINE2 expression is regulated by pro-inflammatory cytokines [12,13] and in the last years, it was suggested a role for SERPINE2 in the regulation of matrix metalloproteinases activity in glioma and endothelial cells, and in the muscle ECM homeostasis [14–16]. In fact, due to its ability to influence the degradation of ECM components, a role for SERPINE2 in cancer cell invasion and metastasis was proposed [17,18]. However, only a few data about the functions of SERPINE2 at cartilage level are known. Stevens et al published the only evidence of the participation of this protein in cartilage ECM homeostasis. These authors demonstrated that SERPINE2 was able to inhibit the glycosaminoglycan loss elicited by intraarticular injection of IL-1 in rabbits [19].

Thus, in this study we aimed to determine the constitutive expression of SERPINE2 in human chondrocytes, and to evaluate its regulation by the pro-inflammatory cytokine IL-1α. Next, we assessed the ability of SERPINE2 to counteract the IL-1α-induced MMP-13 expression. Finally, we explored the signaling pathway involved in this mechanism, focusing on the activation of ERK 1/2 and the transcription factors NF-κB and AP-1.

## Materials and Methods

### Reagents

Fetal bovine serum (FBS), Dulbecco’s modified Eagle’s medium (DMEM)/Ham’s F12 medium, L-glutamine, antibiotics and trypsin-ethylendiaminetetraacetic acid were purchased from Lonza (Switzerland). Human recombinant SERPINE2 was obtained from R&D System (MN, USA), while human recombinant IL-1α was purchased from Sigma (MO, USA). ERK 1/2 pharmacological inhibitor PD098059 was purchased from Sigma (MO, USA). For RT-PCR, a First Strand Kit, Master mix, primers for SERPINE2, MMP-13 and GAPDH were purchased from SABiosciences (MD, USA). Nucleospin kits for RNA isolation were from Macherey-Nagel (Germany).

### Cell cultures

Human primary chondrocytes and T/C-28a2 cell line culture were developed as previously described [20,21]. Briefly, osteoarthritic human cartilage samples were obtained from patients undergoing total joint replacement surgery (this study was conducted with the approval of the Santiago University Clinical Hospital Ethics Committee). Cartilage samples were obtained from the joint area of minimal load with normal morphologic examination (i.e., no change in color and no fibrillation). Human chondrocytes were cultured in DMEM/Ham’s F12 medium supplemented with 10% of fetal bovine serum, L-glutamine, and antibiotics (50 units/ml penicillin and 50 μg/ml streptomycin). Cells were seeded in monolayer up to the high density and used freshly in order to avoid dedifferentiation.

The immortalized human juvenile costal chondrocyte cell line T/C-28a2 (a kind gift from Dr. Mary B. Goldring, Hospital for Special Surgery, NYC, USA) was cultured in DMEM–Ham’s F-12 medium supplemented with 10% FBS, L-glutamine, and antibiotics (50 units/mL penicillin and 50 μg/mL streptomycin).

### mRNA isolation and real-time RT-PCR

For real-time reverse transcription PCR (RT-PCR) studies, cells were seeded in P6 multiwell plates until complete adhesion (85% to 90% confluence) and then incubated overnight in serum-free conditions. Cells were treated with human IL-1α (0.05, 0.5, 1, 5 and 10 ng/mL) or, in another set of experiments, with SERPINE2 (0.4 ng/mL) alone or in presence of IL-1α (0.5 ng/mL) for 24 hours.

Human SERPINE2 or MMP-13 mRNA levels were determined using SYBR Green-based semi-quantitative PCR. RNA was extracted using a NucleoSpin kit (Macherey-Nagel, Dϋren, Germany), according to the manufacturer’s instructions and reverse-transcribed (RT) using a SABiosciences First Strand Kit. After the RT reaction, qPCR analysis was performed with a SABiosciences Master Mix and specific PCR primers for: human SERPINE2 (156 bp, PPH08354A, reference position 1101, GenBank accession no. NM_006216.3); human MMP13 (150 bp, PPH00121B, reference position 221–241, GenBank accession no. NM_002427.2); human GAPDH (175 bp, PPH00150E, reference position 1287–1310, GenBank accession no. NM_002046.3. Amplification efficiencies were calculated for all primers utilizing serial dilutions of the pooled cDNA samples. The data were calculated, using the comparative (ΔΔCt) method and the MxPro software (Stratagene, CA, USA), as the ratio of each gene to the expression of the housekeeping gene. Data are shown as mean ± s.e.m (error bars) of at least three independent experiments and represented as fold-change vs. controls. Melting curves were generated to ensure a single gene-specific peak, and no-template controls were included for each run and each set of primers to control for unspecific amplifications.

### Western blot analysis

To determine SERPINE2 and MMP-13 protein expression, human primary chondrocytes and T/C-28a2 cells were incubated with IL-1α and/or recombinant SERPINE2 for 24 hours. In another set of experiments, to assess ERK1/2 phosphorylation and IκB-α degradation, cells were pretreated with recombinant SERPINE2 (0.4 ng/mL) for 12 hours and then challenged with IL-1α (0.5 ng/mL) for 30 min. In both experiments, after the stimulation, cells were washed twice with ice cold PBS, harvested, and resuspended in lysis buffer for protein extraction (10 mM Tris/HCl, pH 7.5, 5 mM EDTA, 150 mM NaCl, 30 mM sodium pyrophosphate, 50 mM sodium fluoride, 1 mM sodium orthovanadate, 0.5% Triton X-100, 1mM PMSF, protease inhibitor cocktail). Cell lysates were obtained by centrifugation at 14.000xg for 20 min at 4°C. In another set of experiments, to evaluate p65 and c-Jun subunits translocation to the nucleus, T/C-28a2 chondrocytes were pretreated with recombinant SERPINE2 (0.4 ng/mL) for 12 hours and then challenged with IL-1α (0.5 ng/mL) for 15 or 30 min, respectively. Afterwards, cells were subjected to a differential lysis to obtain the nuclear and cytosolic fractions as previously described [22].

Protein concentrations were estimated by the Bio-Rad protein assay using bovine serum albumin as standard. Lysates from control or stimulated cells were collected and separated by SDS/PAGE on a 10% polyacrylamide gel. Proteins were subsequently transferred to a polyvinylidene difluoride transfer membrane (Immobilon-P transfer membrane, Millipore, MA) using a transfer semidry blot cell (BioRad Laboratories). Blots were incubated with the appropriate antibody: anti-human SERPINE2 (R&D, MN, USA); anti-human MMP-13 (Santa Cruz, CA, USA); anti-phospho ERK1/2 (Millipore, MA, USA); anti-ERK1/2 (Millipore, MA, USA), anti-p65 (Santa Cruz, CA,USA); anti-c-jun (Santa Cruz, CA, USA). Immunoblots have been visualized with Immobilon Western Detection kit (Millipore, MA) using horseradish peroxidase-labeled secondary antibody. To confirm equal loading in each sample, the membranes were stripped in stripping buffer (100 mM β-mercaptoethanol, 2% SDS, 62.5 mM Tris-HCl pH 6.7) and re-blotted with anti-β-actin (Sigma, MO, USA) or anti-lamin-β1 antibody (GeneTex, CA, USA). The images were captured and analyzed with an EC3 imaging system (UVP). Densitrometric analyses were performed using ImageJ software (National Institutes of Health, Bethesda, MD, USA).

### Determination of MMP-13 activity

MMP-13 activity in the conditioned medium of OA human primary chondrocytes was measured using a SensoLyte Plus 520 MMP-13 Assay Kit (AnaSpec, CA, USA) following manufacture´s instructions. To obtain the conditioned medium, we treated OA human primary chondrocytes with recombinant SERPINE2 (0.4 ng/mL) or/and IL-1α (0.5 ng/mL) during 24 hours.

### SERPINE2 gene knockdown

For siRNA transfection experiments, T/C-28a2 cells were seeded at 2x10^5^ cells per well in 6-well plates and incubated overnight with DMEM/Ham´s F12 with 10% FBS. The medium was then changed to serum and antibiotics free medium. Transfections were performed following manufacture´s instructions (Integrated DNA Technologies, USA). Gene silencing was made using 10 nM of SERPINE2 siRNA, we also used 10 nM of a non-targeting control to verify the specificity of the SERPINE2 siRNA. Incubation was continued for 48 hours after siRNA tranfection and the SERPINE2 gene knockdown was verified at mRNA and protein levels (data not shown). At 48 hours after transfection, the cells were treated with recombinant SERPINE2 (0.4 ng/mL) or/and IL-1α (0.5 ng/mL) during 24 hours.

### Statistical analysis

Data are reported as mean ± standard error mean (S.E.M.) values of independent experiments, which were done at least three times, each time with three or more independent observations. Statistical analysis was performed by analysis of variance test, and multiple comparisons were made by Bonferroni’s test or, when appropriate, with Dunnet’s test. Statistical significance was set at P<0.05.

## Results

### Effects of IL-1α treatment on human SERPINE2 expression

**Fig 1:**
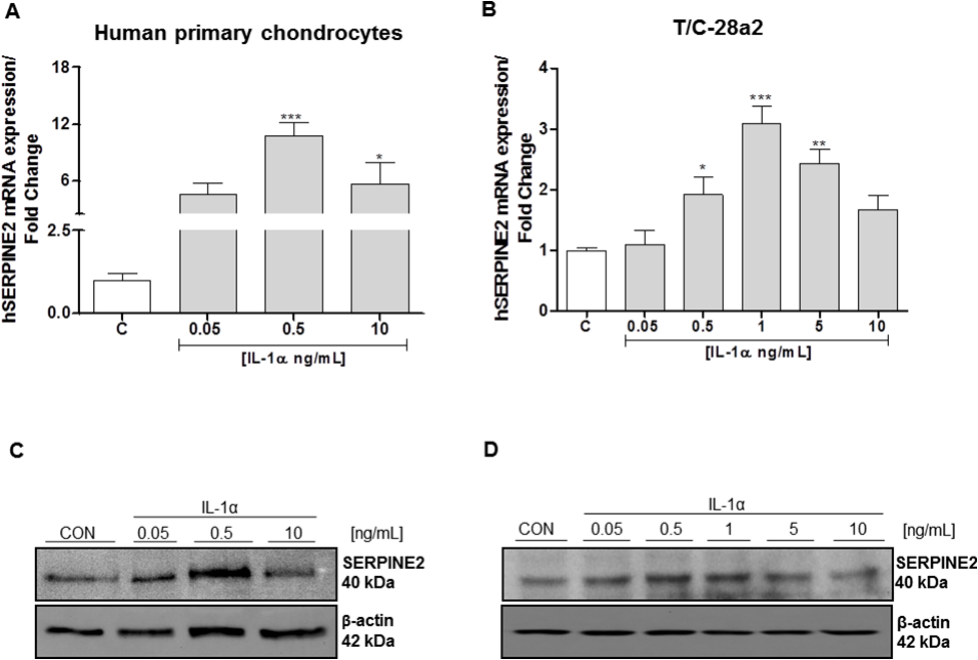
mRNA and protein expression of SERPINE2 were determined by real-time PCR and western blot analysis respectively. **A, B**. Human SERPINE2 mRNA expression after interleukin IL-1α treatment (0.05–10 ng/mL) during 24 hours in human primary chondrocytes and in T/C-28a2 chondrogenic cells. **C, D**. Representative western blot of human SERPINE2 protein expression in lysates obtained from human primary chondrocytes and T/C-28a2 chondrogenic cells treated with interleukin IL-1α (0.05–10 ng/mL) for 24 h. β-actin was used to ensure equal sample loading. Data are means ± S.E.M. of at least 3 independent experiments. *P<0.05, **P<0.01 and ***P<0.001 vs untreated control cells.

As shown in Fig 1, we were able to detect a basal mRNA and protein expression of SERPINE2 in both, human primary chondrocytes and T/C-28a2 cells.

In order to assess the effect of IL-1α on SERPINE2 expression, human primary chondrocytes and T/C-28a2 cells were treated with this pro-inflammatory cytokine at increasing concentrations (0.05–10 ng/mL) for 24 h. As shown in Fig 1A and 1C, IL-1α stimulation, significantly induced SERPINE2 mRNA and protein expression in human primary chondrocytes, reaching the highest levels at dose of 0,5 ng/mL. To note, we observed a similar pattern of expression in T/C-28a2 chondrocytes (Fig 1B and 1D).

### Effect of SERPINE2 on IL-1α –induced MMP-13 expression

**Fig 2:**
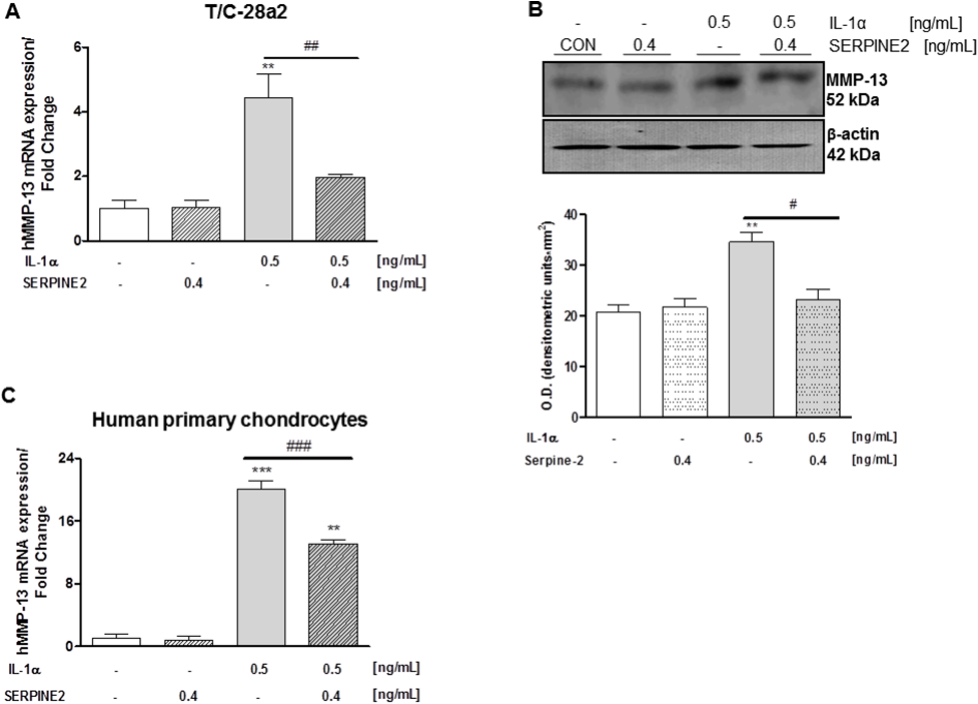
MMP-13 mRNA and protein levels were determined by real-time PCR and western blot analysis respectively. **A.** Human MMP-13 mRNA expression in T/C-28a2 chondrocytes incubated with SERPINE2 (0.4 ng/mL) in presence or not of IL-1α (0.5 ng/mL) for 24 h. **B.** Representative western blot of human MMP-13 protein expression in lysates obtained from T/C-28a2 chondrogenic cells treated with with SERPINE2 (0.4 ng/mL) in presence or not of IL-1α (0.5 ng/mL) for 24 h. β-actin was used to ensure equal sample loading. **Low panel.** Densitometric analysis of at least three independent experiments. **C.** Human MMP-13 mRNA expression in human primary chondrocytes incubated with SERPINE2 (0.4 ng/mL) in presence or not of IL-1α (0.5 ng/mL) for 24 h. Data are means ± S.E.M. of at least 3 independent experiments. **P<0.01 and ***P<0.001 vs untreated control cells; ^##^P<0.01 and ^###^ P<0.001 vs IL-1α-stimulated chondrocytes.

Once determined the modulation of SERPINE2 expression by IL-1α, we sought to analyze whether this protein has a regulatory effect on the expression of one of the most relevant collagenases, MMP-13. As shown in Fig 2A, IL-1α showed a significant up-regulation of MMP-13 mRNA expression after 24h challenge in T/C-28a2 chondrocytes. Interestingly, recombinant SERPINE2 was able to revert, efficiently, the induction of MMP-13 elicited by IL-1α. These results were also confirmed in terms of protein expression (Fig 2B).

We also observed that recombinant SERPINE2 reduced the induction of MMP-13 mRNA expression stimulated by IL-1α in human primary chondrocytes (Fig 2C).

**Fig 3:**
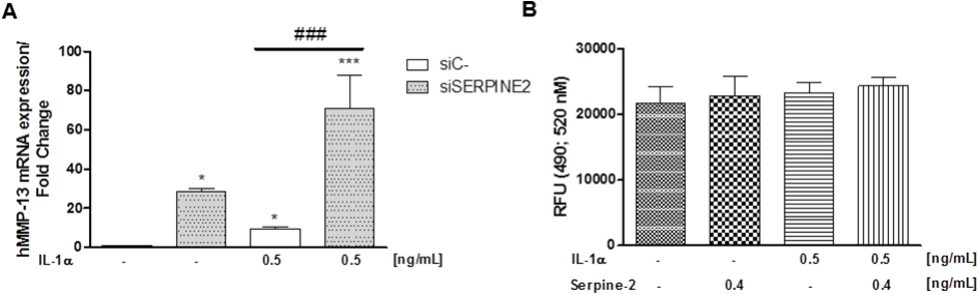
A. Human MMP-13 mRNA expression in T/C-28a2 chondrocytes transfected with a siRNA against SERPINE2 (siSERPINE2, 10nM) or a non-targeting control siRNA (siC-, 10 nM) in presence or not of IL-1α (0.5 ng/mL) for 24 h. B. MMP-13 endogenous catalytic activity was measured in the conditioned medium of OA human primary chondrocytes after recombinant SERPINE2 (0.4 ng/mL) treatment in presence or not of IL-1α (0.5 ng/mL) for 24 h. *P<0.05 and ***P<0.001 vs siC- transfected cells; ^###^ P<0.001 vs siC- plus IL-1α-stimulated chondrocytes.

To address the role of endogenous SERPINE2 on MMP-13 expression we used a different approach. In this case, we silenced the expression of SERPINE2 by using a siRNA against this serine protease. As shown in Fig 3A, SERPINE2 gene knockdown led to an increase in MMP-13 expression in comparison to cells transfected with a non-targeting control siRNA. We also observed that SERPINE2 silencing produced a strong up-regulation of MMP-13 mRNA expression in IL-1α treated chondrocytes (Fig 3A).

For completeness, we also tested whether SERPINE2 had any effect on MMP-13 catalytic activity. As shown in Fig 3B, in our experimental conditions we did not determine any significant variations in the endogenous active form of MMP-13.

### SERPINE2 inhibits ERK1/2, NF-κB and AP-1 signaling pathways

Since MAPKs, NF-κB, and AP-1 pathways were previously found to play a crucial role in inducing MMP-13 expression and cartilage catabolism in chondrocytes [23,24], here we investigated the effects of SERPINE2 on these signaling transduction pathways.

**Fig 4:**
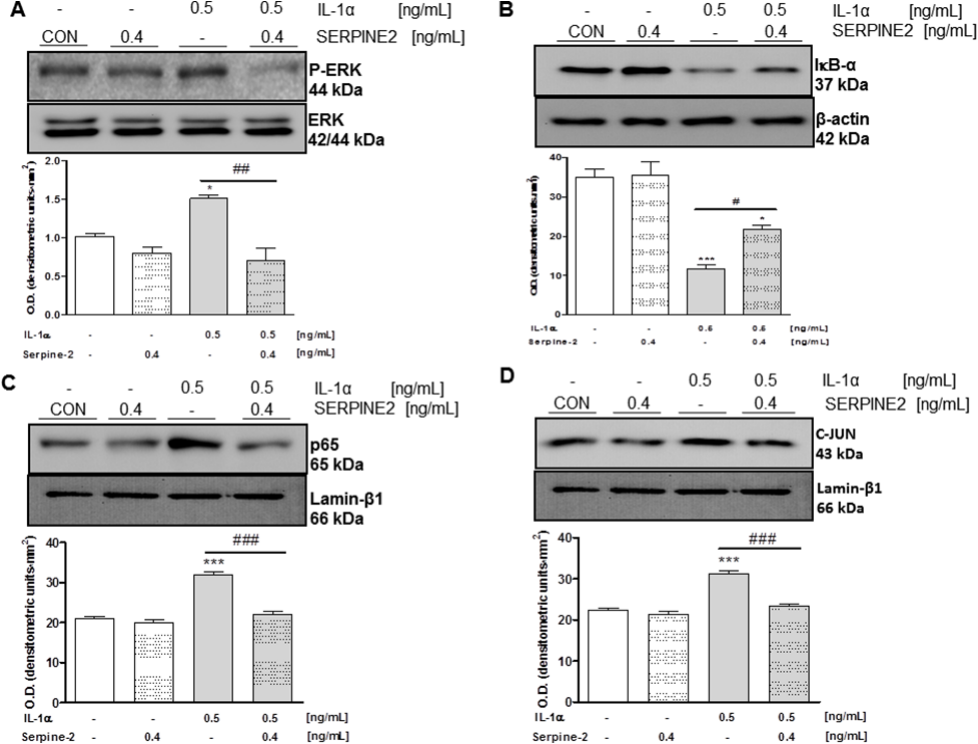
A. Analysis of ERK1/2 phosphorylation by western blot using lysates obtained from T/C-28a2 chondrogenic cells pretreated with SERPINE2 (0.4 ng/mL) for 12 h and then challenged with IL-1α (0.5 ng/mL) for 30 min. Total ERK was used to ensure equal sample loading. B. IκB-α protein expression determined by western blot using lysates obtained from T/C-28a2 chondrogenic cells pretreated with SERPINE2 (0.4 ng/mL) for 12 h and then challenged with IL-1α (0.5 ng/mL) for 15 min. β-actin was used to ensure equal sample loading. C, D. p65 and c-Jun expression determined by western blot using nuclear extract obtained from T/C-28a2 chondrogenic cells pretreated with SERPINE2 (0.4 ng/mL) for 12 h and then challenged with IL-1α (0.5 ng/mL) for 30 min. LaminB1 was used to ensure equal sample loading. **Low panels.** Densitometric analysis of at least three independent experiments. *P<0.05 and ***P<0.001 vs untreated control cells; ^#^P<0.05, ^##^P<0.01 and ^###^ P<0.001 vs IL-1α-stimulated chondrocytes.

As shown in Fig 4A, IL-1α treatment led to an increase in ERK-1/2 phosphorylation, which was completely inhibited by recombinant SERPINE2 addition.

To gain further insights into the mechanism of action underlying the down-regulation of MMP-13 by SERPINE2 treatment, we also investigated the NF-κB signaling pathway. We observed that IL-1α induced a strong IκB-α degradation and interestingly, the addition of recombinant SERPINE2 partially blocked the IκB-α degradation (Fig 4B). In line with this, recombinant SERPINE2 treatment also blocked the translocation elicited by IL-1α of the NF-κB subunit p65 to the nucleus (Fig 4C).

Finally, we also sought to analyze whether SERPINE2 was able to modulate other transcription factor beside NFκB. For this reason, we analyse AP-1. As shown in Fig 4D, and as expected, IL-1α was able to increase the translocation of the AP-1 family member c-jun to the nucleus, and interestingly, the addition of recombinant SERPINE2 reduced the translocation of c-jun exerted by this pro-inflammatory cytokine.

## Discussion

Osteoarthritis is characterized by increased production of matrix-degrading enzymes. Actually, an irreversible step in OA progression occurs when the balance of collagen degradation is perturbed. In this process, collagenases, such as MMP-13, were thought to play a pivotal role. Thus, particular emphasis has been placed in studying the regulation of MMPs expression. In this scenario, the serine protease inhibitor family (serpin) seems to regulate the activity of different MMPs [7,8,14]. However, the participation or function of SERPINE2 in the above mentioned mechanism at cartilage level is at present unknown.

In the present study, we show for the first time, that human chondrocytes express SERPINE2. In addition to basal expression levels, we have demonstrated that IL-1α determines a clear up-regulation of SERPINE2. Our results are consistent with those obtained by other authors [12,13], showing the ability of several injury-related cytokines, such as IL-1 or TNF-α, to induce the expression of SERPINE2 in other cell types, such as cultured brain cells and normal human fibroblasts. Interestingly, our data have shown that the expression pattern of SERPINE2 in response to increasing doses of IL-1α follows a bell-shaped profile. This result is in agreement with other previous data, which demonstrated that, several pro-inflammatory cytokines, including IL-1α and TNF-α, induced SERPINE2 secretion in a concentration-dependent manner [12].

The increased cellular level of SERPINE 2 in response to IL-1 stimulation suggests a compensatory mechanism under catabolic imbalance. So, it is conceivable that an increase in SERPINE2 level represents an attempt to antagonize the well-known pro-inflammatory effect of IL-1, suggesting that these two factors may act in parallel as opposing metabolic counterparts.

Concerning the role of SERPINE2 in preventing ECM degradation, previous findings have demonstrated that this protein suppress the activity of degradative enzymes, such as ADAM metallopeptidase domain 17 (ADAM17), in endothelial vascular cells [15]. The evidence that SERPINE2 is able to strongly decrease the IL-1α-driven MMP13 expression provides new insights on the regulation of MMPs activity in cartilage. To note, MMP-13 is not only a rate-limiting enzyme involved in collagen degradation, it also degrades aggrecan, suggesting a potential dual role in matrix destruction [25,26].

Accordingly, very recently was demonstrated that SERPINE2 gene knockdown increased MMP-9 and MMP-2 expression in C6 glioma cell line [14]. Furthermore, Stevens et al. showed that SERPINE2 reduced cartilage glycosaminoglycan loss produced by intraarticular injection of IL-1 in rabbit [19]. Our study demonstrates that SERPINE2 is able to partially counteract the catabolic effect of IL-1α, by inhibiting MMP-13 induction, suggesting a potential protective role for SERPINE2 by decreasing the collagen breakdown exerted by this metalloproteinase at cartilage level. This aspect may allow the development of drugs and treatments strategies to prevent or limit cartilage breakdown in OA and RA. To gain further insights into the specificity of SERPINE2 on IL-1-induced MMPs expression, we silenced this serine protease with a specific siRNA. In our experimental set, the fact that SERPINE2 silencing (in absence of IL-1 stimulation, see Fig 3A) is able to increase, per se, MMP-13 intracellular expression suggests that the inhibitory activity of SERPINE2 is not related to any “sticky” interaction with IL-1 or its receptor. However, the precise mechanism by which SERPINE2 interacts with intracellular signal transducers is at present unknown and deserves further studies.

To elucidate the key regulators and signaling pathways involved in MMP-13 inhibition by SERPINE2, we first analysed the modulation of ERK-1/2 phosphorylation. Here, we demonstrated that SERPINE2 inhibited ERK-1/2 activation induced by IL-1α. ERK activation drives the induction of downstream transcriptional effectors with direct impact on chondrocyte physiology, as MMP13 [23,27,28]. To note, our results are in agreement with those obtained by Acosta et al [29] and by Pagliara et al [14]. MAPKs, such as ERK 1/2, may activate different downstream transcription factors, for instance NF-κB, which mediates much of the downstream effects of IL-1, including the induction of MMPs and other pro-inflammatory cytokines. The fact that SERPINE2 counteracts IL-1α-driven IκB-α degradation and p65 translocation to the nucleus, suggests that SERPINE2-induced MMP-13 repression could be mediated by the inhibition of ERK 1/2 and by the downstream transcription factor NF-κB. This mechanism is likely to be exerted also on c-jun.

In conclusion, our results highlight the relevance of SERPINE2 in chondrocyte pathophysiology. Its inhibitory effect on MMP-13, one of the most relevant collagenases involved in extracellular matrix degradation and articular cartilage destruction, suggests that SERPINE2 may have beneficial effects in joint degenerative diseases, such as OA, and should be considered as a potential therapeutic target useful in this pathology. In addition, our data suggest that SERPINE2 may regulate intracellular kinases activity and transcription factors that are at play in the regulation of MMPs expression. Taken together, all these results make SERPINE2 an attractive pharmacologic target. However questions still remain to be answered to understand the precise role of SERPINE2 in the regulation of the mechanism involved in the development and or progression of degenerative-inflammatory joint diseases.
